# Structure–Property Relationships in Epoxy–Anhydride Systems: A Comprehensive Comparative Study of Cycloaliphatic, Novolac, and Aromatic Prepolymers

**DOI:** 10.3390/polym17212843

**Published:** 2025-10-24

**Authors:** Stephane Patry, Alban Asseray, Mickaël Berne, Valéry Loriot, Luc Loriot, Jean-Pierre Habas

**Affiliations:** 1 ICGM, Institut Charles Gerhardt, Univ Montpellier, CNRS, ENSCM, 34000 Montpellier, France; stephane.patry@umontpellier.fr; 2 Resoltech, 249 Avenue Gaston Imbert ZI DE, 13790 Rousset, France; a.asseray@resoltech.com (A.A.); m.berne@resoltech.com (M.B.); v.loriot@resoltech.com (V.L.); l.loriot@resoltech.com (L.L.)

**Keywords:** epoxy prepolymers, anhydride hardener, reactivity, glass transition temperature, thermal stability, rheology, crosslink density, structure-property relationships, thermal aging resistance

## Abstract

This study provides a comprehensive quantitative comparison of three structurally distinct epoxy prepolymers—cycloaliphatic, novolac, and bis-aromatic (BADGE)—cured with a single hardener, methyl nadic anhydride (MNA), and catalyzed by 1-methylimidazole under strictly identical stoichiometric and thermal conditions. Each formulation was optimized in terms of epoxy/anhydride ratio and catalyst concentration to ensure meaningful cross-comparison under representative cure conditions. A multi-technique approach combining differential scanning calorimetry (DSC), dynamic rheometry, and thermogravimetric analysis (TGA) was employed to jointly assess cure kinetics, network build-up, and long-term thermal stability. DSC analyses provided reaction enthalpies and glass transition temperatures (*Tg*) ranging from 145 °C (BADGE-MNA) to 253 °C (cycloaliphatic ECy-MNA) after stabilization of the curing reaction under the chosen thermal protocol, enabling experimental fine-tuning of stoichiometry beyond the theoretical 1:1 ratio. Isothermal rheology revealed gel times of approximately 14 s for novolac, 16 s for BADGE, and 20 s for the cycloaliphatic system at 200 °C, defining a clear hierarchy of reactivity (Novolac > BADGE > ECy). Post-cure thermomechanical performance and thermal aging resistance (100 h at 250 °C) were assessed via rheometry and TGA under both dynamic and isothermal conditions. They demonstrated that the novolac-based resin retained approximately 93.7% of its initial mass, confirming its outstanding thermo-oxidative stability. The three systems exhibited distinct trade-offs between reactivity and thermal resistance: the novolac resin showed superior thermal endurance but, owing to its highly aromatic and rigid structure, limited flowability, while the cycloaliphatic resin exhibited greater molecular mobility and longer pot life but reduced stability. Overall, this work provides a comprehensive and quantitatively consistent benchmark, consolidating stoichiometric control, DSC and rheological reactivity, *Tg* evolution, thermomechanical stability, and degradation behavior within a single unified experimental framework. The results offer reliable reference data for modeling, formulation, and possible use of epoxy–anhydride thermosets at temperatures above 200 °C.

## 1. Introduction

The continuous processing of fiber-reinforced polymer composites is a highly efficient and scalable method for producing structural components with constant cross-sectional profiles. Its high productivity and dimensional reproducibility make it particularly attractive for applications in construction, transportation, electrical insulation, and renewable energy. However, the performance and cost-effectiveness of such composites are strongly governed by the formulation and physicochemical behavior of the thermosetting resin system used as the matrix.

Among thermosetting polymers, epoxy resins remain the preferred choice in many high-performance composite applications due to their mechanical strength, low shrinkage, chemical and corrosion resistance, and high thermal stability [1]. Nevertheless, the selection of an appropriate curing system, including the type of hardener and catalyst, as well as the choice of epoxy prepolymer, significantly influences not only the final properties of the composite but also its processability and manufacturing control [2].

Anhydride-cured epoxy systems offer several advantages over amine-cured analogs for continuous processing and high-temperature applications. Their curing reactions are less exothermic and more controllable, typically providing longer pot lives and wider processing windows [3,4]. The resulting polymer networks exhibit higher glass transition temperatures (*Tg*), lower moisture uptake, and improved dielectric properties, which are highly desirable for structural and electrical insulation applications [5,6,7,8]. Despite these advantages, epoxy–anhydride systems remain challenging to formulate as their performance depends strongly on the molecular structure of the epoxy prepolymer and on the stoichiometric balance of reactive groups [9,10].

Although the mechanisms of epoxy–anhydride reactions have been widely investigated, comparative studies performed under strictly identical catalytic and stoichiometric conditions remain limited [1,2,11,12]. Most reported works either focus on a single epoxy type or vary several formulation parameters simultaneously (e.g., hardener type, catalyst, or stoichiometric ratio), which makes it difficult to isolate the specific influence of the epoxy prepolymer structure. In addition, many studies emphasize macroscopic performance or predictive modeling rather than detailed experimental correlations between molecular architecture, cure kinetics, and thermal behavior.

This lack of integrated, directly comparable datasets represents a key limitation in understanding how epoxy molecular structure governs anhydride-curing behavior. Previous investigations often rely on a limited number of characterization techniques, which hinder consistent cross-validation of thermal, rheological, and thermo-stability data. Yet, the combination of complementary methods is essential to distinguish kinetic effects from physical transitions and to establish reliable structure–property relationships.

The present work therefore aims to bridge this gap by conducting a systematic and quantitatively coherent comparison of three chemically distinct epoxy prepolymers under identical stoichiometric and catalytic conditions. By integrating thermal and rheological analyses within a unified experimental protocol, this study provides a consistent framework to correlate molecular architecture with cure reactivity, glass transition development, and thermal endurance at the matrix level.

Building on this comparative framework, the next step was to define the formulation parameters ensuring meaningful and reproducible comparisons among the different epoxy–anhydride systems. Considering practical aspects, one of the key formulation challenges lies in reducing system complexity and the number of raw materials involved, without compromising performance. The ability to use a single industrial anhydride hardener with different classes of epoxy prepolymers—while maintaining adequate cure behavior, processability, and thermal resistance—would represent a significant practical advantage, simplifying formulation design and improving reproducibility.

In this context, methyl nadic anhydride (MNA) was selected as the reference hardener because it combines high reactivity and excellent compatibility with the three epoxy families considered—cycloaliphatic, novolac, and bisphenol-A-based (BADGE). MNA is a liquid anhydride, which simplifies handling and avoids sedimentation problems that may arise with solid hardeners (such as nadic or phthalic). Compared to other liquid anhydrides such as methylhexahydrophthalic (MHHPA) or methyltetrahydrophthalic (MTHPA) anhydrides, MNA possesses a rigid bicyclic structure that promotes the formation of crosslinked materials with enhanced ultimate properties. Its low volatility and moderate exothermicity make it particularly suitable for producing matrices with long-term thermal stability above 200 °C. From a practical standpoint, its wide commercial availability from multiple suppliers ensures formulation reproducibility and material consistency.

The choice of an anhydride rather than a polyamine hardener was guided by processing considerations, since anhydrides offer slower reactivity and longer pot life at 25–60 °C, preventing premature gelation during impregnation or mixing while still enabling high *Tg* values after curing [11,13,14]. In this study, the resin design targeted a viscosity below approximately 1000–1500 mPa·s between 60 °C and 120 °C, a range commonly used to ensure proper wetting and homogeneous flow during composite fabrication. This formulation strategy provided the foundation for the present comparative investigation.

Although the present work focuses exclusively on neat resin systems, it constitutes a fundamental prequalification step, establishing the quantitative basis required before any possible integration of fillers or fibers. A detailed understanding of reactivity, processing windows, and thermal stability at the matrix level is essential to ensure reliable behavior under sustained exposure to elevated temperatures.

To this end, the present work conducts a systematic and quantitatively consistent comparison of three chemically distinct epoxy prepolymers cured with the same anhydride hardener, focusing on their reactivity, thermomechanical properties, and thermal endurance. The prepolymers investigated include three types of epoxy molecules, namely cycloaliphatic, novolac epoxy, and diaromatic (BADGE), which differ in functionality (with a minimum value of 2), and molecular architecture, factors expected to influence viscosity, cure kinetics, and the properties of the resulting networks.

A comprehensive experimental approach, combining differential scanning calorimetry (DSC), isothermal or dynamic rheology, and thermogravimetric analysis (TGA), was employed to characterize the formulation from mixing to post-cure performance. The influence of the anhydride-t-epoxy ratio and catalyst concentration was systematically optimized to ensure representative and comparable cure conditions. Rheological measurements provided gelation and vitrification times essential for defining practical processing windows, while thermomechanical and thermogravimetric analyses assessed the cured networks’ thermal endurance.

This combination of thermal and rheological techniques enables a robust structure-property correlation, ensuring that observed differences are solely attributable to the structure of the epoxy prepolymer since all other parameters (anhydride, catalyst, and experimental conditions) were kept constant. Rather than introducing new mechanisms, this work aims to consolidate and cross-validate multiple complementary analyses—stoichiometric control, DSC and rheological reactivity, *Tg* evolution, thermomechanical stability, and TGA degradation—within a single, unified experimental framework. Beyond its academic interest, this study provides quantitative benchmark data useful for the design and modeling of epoxy–anhydride systems intended for applications requiring long-term stability above 200 °C.

## 2. Materials and Methods

### 2.1. Chemicals

Three commercially available epoxy prepolymers with distinct chemical structures were selected in this study (Table 1). The cycloaliphatic epoxy prepolymer, also known as 3,4-epoxycyclohexylmethyl-3,4-epoxycyclohexane carboxylate (CAS 2386-87-0) with an epoxy equivalent weight EEW of 126.2 g/eq, was purchased from Jiangsu Tetra New Material Technology Co., Ltd., Shanghai (China). Its molecular structure consists of two substituted cyclohexane rings connected through an ester linkage, each bearing an oxirane ring at the 3,4-positions. This configuration provides a rigid, saturated alicyclic framework and two reactive sites per molecule (difunctional system). The novolac epoxy resin (CAS 28064-14-4) with an EEW of 175 g/eq was purchased from LEUNA Company, Harze (Germany). It is an oligomeric multifunctional resin derived from phenol–formaldehyde repeating units, exhibiting an average epoxy functionality of about 2.5 and a relatively narrow degree-of-polymerization distribution. The presence of pendant glycidyl groups on the aromatic backbone allows the formation of highly crosslinked and thermally resistant networks. The aromatic epoxy resin is represented by bisphenol A diglycidyl ether, also known as 4,4′-isopropylidenediphenol diglycidyl ether (CAS 1675-54-3). It was furnished by Olin, Stade, Germany, (Europe) under the D.E.R 332 grade and has an EEW of 175 g/eq. This difunctional prepolymer contains two glycidyl ether groups attached to aromatic rings, separated by an isopropylidene bridge, which confers high stiffness and chemical resistance while maintaining a moderate viscosity.

All epoxy prepolymers were supplied with purities exceeding 98% *w*/*w* according to the manufacturers’ specifications. These purity levels are representative of standard commercial grades commonly used for thermosetting resin applications.

Methyl nadic anhydride (MNA), a mixture of methyl isomers of methylbicyclo [2.2.1] hept-5-ene-2,3-dicarboxylic anhydride (CAS 25134-21-8), was used as a curing agent. It was provided by Dixie Chemical, Pasadena, CA, (USA) and is characterized by an anhydride equivalent weight AEW of 178.2 g/eq. MNA is a liquid anhydride combining high reactivity and low volatility, which facilitates homogeneous mixing and prevents sedimentation during resin preparation. 1-Methylimidazole (1MI) (CAS 616-47-7) was chosen as a catalyst due to its high efficiency in promoting the ring-opening reaction between epoxy and anhydride groups [15]. It was premixed with MNA before incorporation into the epoxy prepolymer. It was purchased from TCI Europe N.V., Haven (Genk, Belgium). All these chemicals were used without any prior purification.

### 2.2. Preparation of Reactive Formulations

Each reactive formulation was based on the association of one type of epoxy prepolymer with MNA. The MNA quantity *m_A_* (expressed in g) was calculated using the following Formula (1):

(1) $$m_{A}\;={\;m}_{E}\;\times \;\frac{\mathrm{AEW}}{\mathrm{EEW}}\;\times \;r$$

*m_E_* represents the quantity of epoxy prepolymer (in g), and *r* is the ratio of anhydride equivalent to epoxide equivalent, ranging from 0.7 to 1.2.

For all formulations, the catalyst was dosed in weight percentage (% *w*/*w*) of the anhydride quantity. The catalyst 1-methylimidazole (1MI), being a low-viscosity liquid fully miscible with methyl nadic anhydride (MNA), was first premixed with the anhydride under gentle magnetic stirring (approximately 300 rpm) for 2–3 min at room temperature to ensure uniform dispersion before incorporation into the epoxy prepolymer.

For clarity, the formulations are hereafter designated as ECy–MNA, BADGE–MNA, and Novo–MNA, corresponding to the cycloaliphatic, bisphenol-A, and novolac epoxy prepolymers, respectively, cured with methyl nadic anhydride (MNA) and catalyzed with 1-methylimidazole (1MI). The catalyst concentration, expressed as weight percentage relative to the anhydride, is specified in each experiment or table as appropriate.

### 2.3. Preparation of Cured Samples

The cured samples were prepared by pouring the corresponding initial reactive liquid mixture into an aluminum mold (diameter 40 mm) and setting them in a thermostated oven. The curing cycle consisted of 3 h at 80 °C, followed by 60 min at 120 °C, 180 min at 180 °C, and finally, 10 min at 250 °C. Although this thermal schedule is longer than typical process cycles, the initial moderate-temperature steps were chosen to avoid thermal runaway and ensure uniform curing. The risk of thermal gradient or overheating is minimized under laboratory conditions, given the moderate sample thickness. This curing schedule was designed to provide complete conversion and stable network formation under controlled laboratory conditions, rather than to reproduce any industrial thermal profile. The corresponding in-process curing kinetics were evaluated separately under isothermal conditions, as discussed in Section 3.3.1.

### 2.4. Differential Scanning Calorimetric Experiments (DSC)

The calorimetric profile of each freshly prepared reactive formulation, as described previously, was recorded as a function of temperature (*T*) using a differential scanning calorimeter (STARe DSC1 from Mettler Toledo, Colombus, OH, USA) under a constant nitrogen flow of 50 mL/min. Before first use, the DSC calibration was performed using indium as a reference. Then, the same rigorous handling protocol was consistently applied to all the formulations studied in this work. A formulation quantity of 10 mg was placed in an aluminum pan. Then, the capsule was closed with a pre-punctured lid to prevent accidental opening during analysis due to the overpressure effect caused by heating. After filling and sealing, the capsule was immediately placed in the instrumented thermal cell of the DSC apparatus, which was previously set at room temperature. Then, the calorimetric analysis was performed up to 300 °C with a heating rate *β* of 20 °C/min to investigate the reaction domain, as indicated by the presence of an exothermic peak characteristic of the crosslinking reaction. After normalizing the heat flux to the sample weight, the integration of the area of this peak was used to evaluate the reaction enthalpy Δ*H* of each formulation. A second run was performed on the same sample, from 25 °C to 300 °C, to determine the glass transition temperature (*Tg*) of each cured material, following the requirements of ISO 11357-2 standards [16].

### 2.5. Viscosimetric Measurements

Viscosity measurements of the epoxy prepolymers were carried out using an AR2000Ex rheometer from TA Instruments^®^, New Castle, DE, (USA), equipped with a Peltier temperature control system and a Couette concentric cylinder geometry (ϕ_cup_ = 27 mm and ϕ_bob_ = 20 mm). The temperature-dependent viscosity was measured from 25 °C to 140 °C using a heating ramp of 2 °C/min. All measurements were performed under steady shear at a constant shear stress of 0.5 Pa.

### 2.6. Dynamic Rheometry

The first rheological analyses consisted of measuring the kinetics of crosslinking reactions for each freshly prepared reactive formulation. These rheological kinetics were performed using a stress-imposed dynamic rheometer (MCR 102 from Anton Paar^®^, Graz, Austria) equipped with an environmental testing chamber, allowing measurements to be conducted under precise temperature control. For these types of experiments, the rheometer was fitted with a cup–plate geometry suitable for characterizing the evolution of a substance from the liquid to the solid state. The inner diameter of the cup (lower part) was ϕ_cup_ = 45 mm, while the upper plate was chosen to be smaller (ϕ_plate_ = 8 mm) to prevent undesirable side effects.

Before each analysis, the same procedure as detailed below was strictly followed. Firstly, the testing geometry was heated in the rheometer oven at the desired analysis temperature. Once the thermal equilibrium was reached, the freshly prepared reactive mixture, as described in part 2.2, initially in liquid form, was poured into the cup. Next, the upper plate was lowered until it made contact with the upper surface of the sample. The average gap was about 3 mm, and its choice was based on preliminary optimization to ensure a representative resin volume and minimize artefacts associated with thin-layer configurations. Using smaller gaps may lead to underestimated gel times or even partial loss of contact with the upper plate due to sample contraction during the curing process. This geometry therefore provides a good compromise between minimizing edge effects and slippage while maintaining torque sensitivity throughout the liquid-to-solid transition. The kinetic experiment was conducted under a constant stress of 500 Pa and a fixed angular frequency (*ω* = 1 rad s^−1^). The evolution of the complex shear modulus, G* = *G*′ + j *G*″, was monitored as a function of curing time to observe and quantify the progressive transformation of the reactive mixture from the liquid to the solid state. The real component *G*′ is usually named “storage modulus” and is specific to the elastic contribution of the sample, i.e., proportional to the mechanical rigidity of the polymer. The imaginary part *G*″ is named “loss modulus” and relates to the dissipated mechanical energy. When the polymer formulation is in the liquid state, the value of the loss modulus *G*″ is higher than the G′ value. But, in the gel and glassy states, the elastic character is predominant (*G*′ > G″). The gelation time was measured at the point of divergence of the two modulus values as a function of time, as this allows for easier comparison between different formulations than other methods based on multi-frequency experiments [17,18]. By contrast, the vitrification time was defined as the point at which the moduli become almost constant [19].

The second series of rheological analyses consisted of determining the thermomechanical profile of each cured formulation. To this end, the cured samples were prepared according to the procedure detailed in Section 2.3 and were cut into samples of parallelepipedic shape. Typical dimensions of the specimens were 45 mm × 10 mm × 1 mm. The analyses were performed using a stress-controlled dynamic rheometer (AR2000Ex from TA Instruments^®^, New Castle, DE, USA) equipped with a rectangular torsion geometry. The thermomechanical tests were conducted at a heating rate of 3 °C min^−1^ from −150 °C to 300 °C. The strain was fixed at 0.08% after prior investigation of the domain characteristic of the linear rheological behavior. The shearing oscillating angular frequency was kept constant (ω = 1 rad/s). Under this latter condition, the temperature *T_α_*, taken at the maximum of the main relaxation peak on the G″ curve, can be considered as a reliable evaluation of the glass transition temperature (*Tg*) of the cured polymer.

### 2.7. Thermogravimetric Analyses

The thermo-oxidative stability of cured samples, prepared according to the procedure detailed in § 2.3, was examined using a TGA2 thermogravimetric analyzer from Mettler Toledo^®^. The dynamic experiments consisted of measuring the sample’s weight loss under airflow (50 mL min^−1^) as a function of temperature *T* from ambient to 900 °C. The thermal ramp was set at a fixed rate *β* of 5 °C/min. Each measurement was performed on approximately 200 ± 10 mg of sample placed in open alumina crucibles. This relatively high sample mass, fully compatible with the high-capacity design of the instrument, was chosen to ensure representative bulk behavior and minimize possible local inhomogeneities. The degradation temperature was defined as the temperature at which the sample’s residual weight reached approximately 95% of its initial value (named *T*_10_). The same technique was employed in isothermal mode to evaluate the stability of the different cured materials for 100 h at a temperature of 250 °C.

## 3. Results and Discussion

The following results provide a coherent quantitative comparison of structurally distinct epoxy–anhydride systems, without attempting to propose new reaction mechanisms.

### 3.1. Optimization of the Stoichiometry of Different Formulations Using DSC

#### 3.1.1. Investigation of the Effects Produced by the Anhydride/Epoxy Ratio

Differential scanning calorimetry (DSC) experiments were carried out in this study to investigate the effects of varying the anhydride/epoxy ratio on the cure behavior of each reactive formulation. For each epoxy system—cycloaliphatic, novolac, and aromatic—several formulations were prepared by varying the ratio *r* in a controlled manner between 0.7 and 1.2, while keeping the catalyst content constant (i.e., 5% *w*/*w* of the anhydride quantity). During the first DSC run, the occurrence of the crosslinking reaction is verified by the presence of a single dominant exothermic peak whose amplitude and position depend on the exact chemical composition of the formulation. The return of the DSC signal to baseline is interpreted here as the attainment of a stable state under the applied thermal conditions, corresponding to a high degree of advancement of the crosslinking reaction. However, this stage does not necessarily imply that the network is fully cured, as residual functionalities may persist beyond the experimental window.

**Figure 1:**
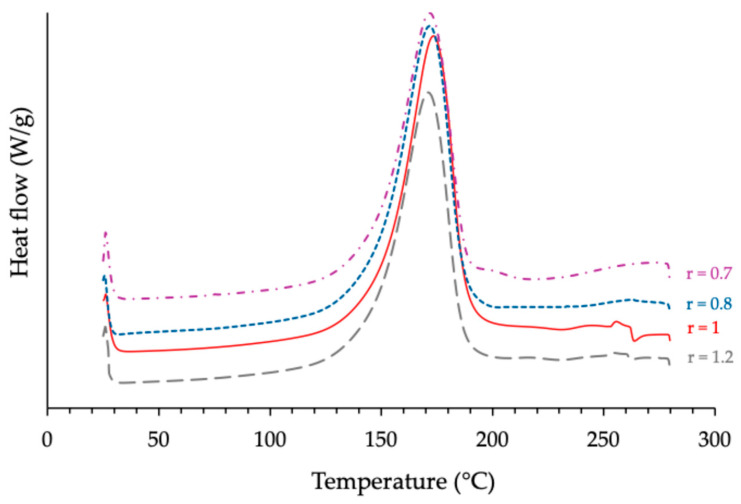
Influence of the anhydride/epoxy ratio *r* on the calorimetric behavior of reactive formulations based on cycloaliphatic prepolymer mixed with anhydride hardener and 5% *w*/*w* of 1MI.

**Figure 2:**
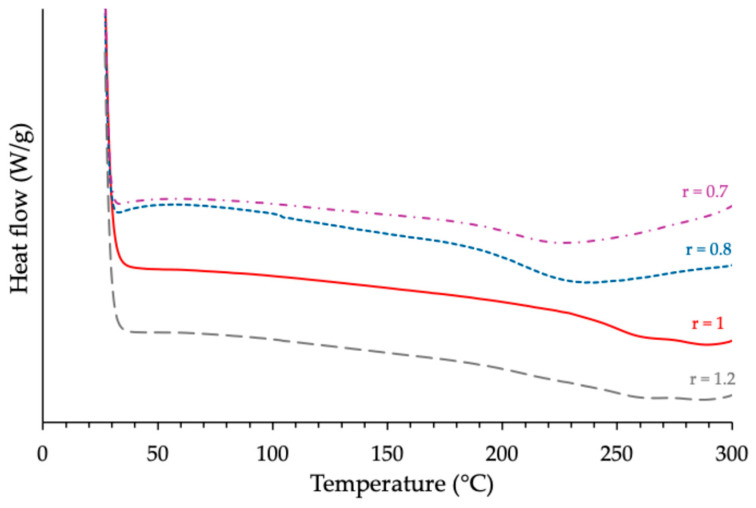
Effects of the epoxy/anhydride ratio on the glass transition temperature of the cured ECy-MNA-1MI 5% *w*/*w* as measured by DSC.

Consecutive DSC analyses of each cross-linked sample revealed an inflection on the corresponding thermogram, which is known to be characteristic of the glass transition phenomenon of the material studied. Figure 1 and Figure 2 show, as examples, the results obtained with the formulations based on the use of ECy as an epoxy prepolymer, during the first and second runs, respectively.

For each reactive formulation, the peak temperature (*Tp*) and the total reaction enthalpy (Δ*H*), normalized to sample mass, were extracted from the DSC thermograms recorded during the first experiment series. The glass transition temperature (*Tg*) of each cured material was determined from the second heating run. The corresponding data are summarized in Table 2. Examination of these results reveals that the optimal anhydride/epoxy molar ratio *r* depends somewhat on the prepolymer structure. For the system based on cycloaliphatic epoxy (ECy), both Δ*H* and *Tg* reached their maximum values at *r* = 1, in agreement with the theoretical stoichiometry. In contrast, the novolac- and BADGE-based formulations displayed slightly higher Δ*H* values at epoxy-rich conditions (r ≈ 0.7–0.8). However, this coincided with only marginal changes in *Tg*, never exceeding about 5 °C relative to stoichiometry.

This apparent discrepancy—higher reaction enthalpy but slightly lower *Tg*—reflects the distinction between overall reaction conversion and crosslinking efficiency. While Δ*H* reflects the total heat released by chemical reactions, *Tg* is primarily determined by the density and effectiveness of the crosslinking network. Under epoxy-rich conditions, additional reactions may occur. On the one hand, epoxy homopolymerization, particularly in the presence of imidazole catalysts, contributes extra exothermicity to the DSC signal, thereby raising the apparent Δ*H*. On the other hand, the imbalance in reactive groups favors the formation of chain ends or pendant structures that consume functional groups without contributing to crosslink junctions. The combined effect is that more heat is released overall, but the resulting network is less efficiently crosslinked, leading to a slight decrease in *Tg*.

Taken together, these results indicate that, despite minor formulation-specific shifts, *r* = 1 remains a robust and practical reference point for all three systems. This stoichiometric ratio was therefore retained for subsequent analyses (catalyst content, rheological kinetics, thermomechanical performance), as it provides a consistent reference condition. This choice also mirrors common formulation strategies in practice, where uniform mixing ratios simplify processing and minimize error sources.

#### 3.1.2. Influence of the Catalyst Content

After establishing the optimal anhydride-to-epoxy ratio for the three resin systems, the study next examined the effects of catalyst concentration on the cure behavior of the different reactive formulations, but also on their thermal performance after curing. Then, 1-methylimidazole catalyst—chosen for its efficiency in initiating the ring-opening polymerization of anhydrides—was added at varying weight percentages relative to the mass of anhydride: 1%, 2.5%, 5%, and 7.5% *w*/*w*. By exploiting the trends identified in the previous section, the anhydride/epoxy ratio was kept constant for all tests (*r* = 1).

**Figure 3:**
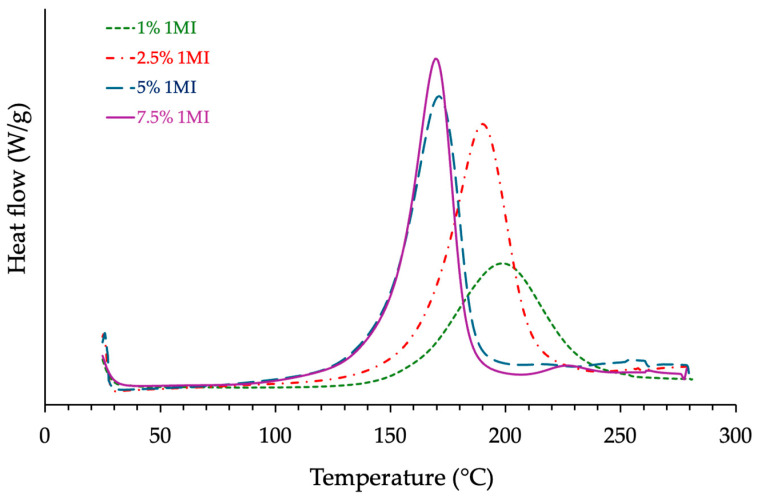
Influence of the catalyst content on the reactivity of the fresh formulation based on ECy prepolymer mixed with MNA hardener (*r* = 1) as measured by DSC.

Dynamic DSC scans were conducted on each formulation under identical conditions (i.e., between 25 °C and 280 °C with a heating rate of 20 °C/min and under nitrogen flow). From the first DSC runs, the total reaction enthalpy (Δ*H*) increased with catalyst content for all systems, reaching its highest values at 7.5% *w*/*w*, consistent with accelerated conversion kinetics. An example is shown in Figure 3 with the ECy-MNA-based formulations. Table 3 provides numerical values of the thermal parameters of Δ*H* and *Tg* as a function of catalyst content for each resin system.

Beyond the mass-normalized values, Table 3 also reports enthalpies per reactive equivalent (Δ*H* in kJ/eq). Interestingly, despite the structural differences between the three epoxy prepolymers, the enthalpy values per equivalent fall within the same range of about 100–120 kJ/eq. This observation indicates that the intrinsic heat released per epoxy–anhydride reaction is essentially similar across all systems. The variations observed in J/g therefore mainly reflect differences in equivalent weights (EEW, AEW) rather than fundamental changes in reaction energetics. In other words, the enthalpy per equivalent is a helpful metric to confirm that the curing chemistry remains comparable across different epoxy structures, and that differences in *Tg* and thermomechanical performance must instead be attributed to network topology and crosslink density rather than to the reaction energetics themselves.

**Figure 4:**
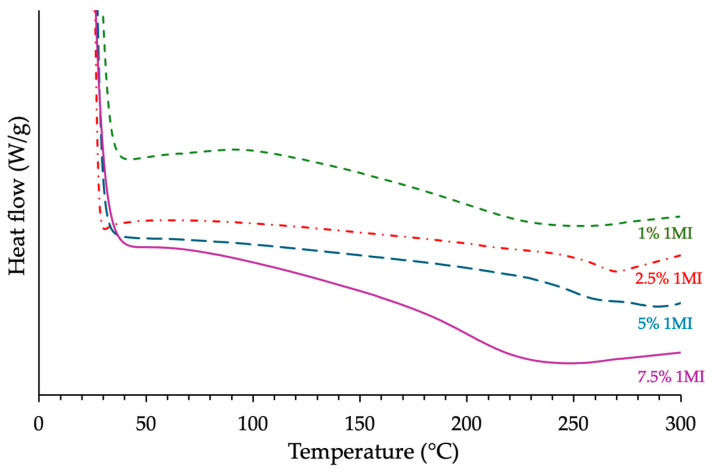
Influence of the catalyst content 1MI on the *Tg* of the cured formulation based on ECy prepolymer mixed with MNA (*r* = 1) as measured by DSC (the curves have been shifted along the vertical axis for clarity).

The second DSC runs, used to evaluate the glass transition temperature (*Tg*) of the cured materials, revealed different trends depending on the epoxy prepolymer (Figure 4 and Table 3). For the BADGE- and ECy-based systems, *Tg* values were lowest at 1% *w*/*w* catalyst, consistent with the reduced reaction enthalpies recorded at this loading. A significant increase in *Tg* was observed at 2.5% *w*/*w*, which corresponded to the most favorable balance between cure conversion and network development. Beyond this value, *Tg* decreased, with a marked drop at 7.5% *w*/*w* for the ECy-based formulation, suggesting that excessive catalyst content may promote side reactions or premature vitrification.

By contrast, the novolac-based system exhibited its highest *Tg* already at 1% *w*/*w* catalyst, with further increases in imidazole content leading to a noticeable decrease. This behavior can be explained by the high functionality of the novolac resin, which allows efficient crosslinking even at low catalyst levels. At higher concentrations, the acceleration of the cure may induce vitrification earlier during the heating ramp, thereby restricting late-stage reactions that are essential for optimal network development. In addition, larger amounts of catalyst can favor epoxy homopolymerization, which increases the apparent Δ*H* without contributing to effective crosslinking, and residual imidazole may act as a local plasticizer, with both effects contributing to a slight *Tg* decrease [20]. All these observations remind us of the dual role of the catalyst: while it promotes epoxy–anhydride reactivity, excessive amounts can compromise the structural integrity of the cured resin [21].

**Table 3:** Thermal parameters extracted from the analyses of the first and second DSC thermograms characteristic of the different formulations, catalyzed with varying weight percentages of 1MI.

Formulation	*x*	*Tp* (°C)	Δ*H* (J/g)	Δ*H* (kJ/eq) *	*Tg* (°C)
ECy-MNA-1MI x% *w*/*w*	1 2.5 5 7.5	199 191 173 169	268 378 329 389	81.6 115 100.1 118.4	178 260 250 199
Novo-MNA-1MI x% *w*/*w*	1 2.5 5 7.5	179 166 156 152	300 330 354 305	106 116.5 125 107.7	209 191 178 167
BADGE-MNA-1MI x% *w*/*w*	1 2.5 5 7.5	183 172 159 159	279 328 320 324	98.5 115.8 113 114.4	157 169 148 145

*: Δ*H* (kJ/eq) is calculated by Δ*H* (J/g) × (*r* × *AEW* + *EEW*)/1000 [1,22].

Although 1% *w*/*w* yielded the highest *Tg* for the novolac formulation, the differences relative to 2.5% *w*/*w* remained limited. From a practical formulation standpoint, however, such a low catalyst content may be considered risky. Indeed, if the catalyst is not perfectly dispersed at the microscopic scale, local under-catalyzed regions could persist, leading to incomplete curing and inconsistent material performance. In contrast, 2.5% *w*/*w* represented the true optimum for both ECy- and BADGE-based systems, providing the highest *Tg* values while avoiding the drawbacks observed at 7.5% *w*/*w*. For these reasons, and to establish a uniform and practically relevant basis for further comparisons, the catalyst concentration was fixed at 2.5% *w*/*w* for all subsequent rheological kinetic, thermomechanical, and thermal stability studies.

Based on these optimizations, all subsequent comparative analyses were performed under fixed conditions: MNA as the standard hardener, stoichiometric ratio *r* = 1, and 2.5% *w*/*w* 1-methylimidazole relative to the anhydride. These parameters ensure that any further differences observed in curing kinetics, glass transition, or thermal stability originate solely from the molecular structure of the epoxy prepolymer.

### 3.2. DSC Study of the Effects Produced by the Epoxy Prepolymer (r = 1 and 2.5% w/w 1MI)

**Figure 5:**
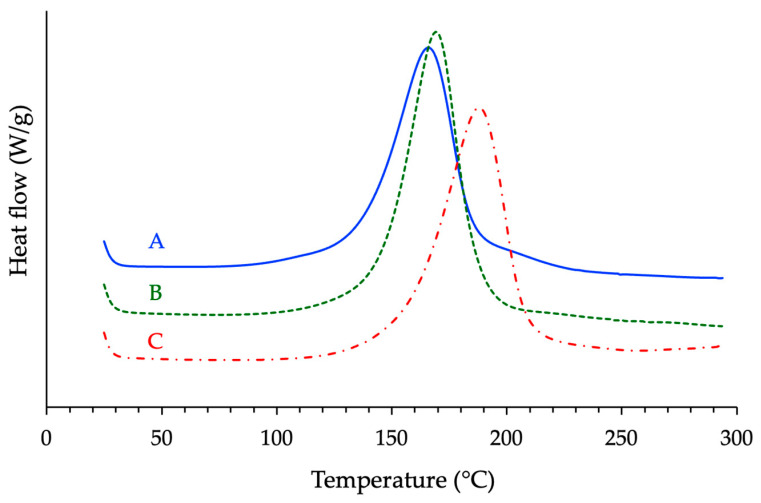
Comparative DSC thermograms of the optimized formulations (A = Novo–MNA, B = BADGE–MNA, C = ECy–MNA) showing distinct exothermic behaviors.

Having established the optimal catalyst loading at 2.5% *w*/*w* relative to the anhydride content, the next phase of the study focused on assessing how the chemical structure of the epoxy prepolymer can influence the reactivity and final thermal properties of the formulations. Differential Scanning Calorimetry (DSC) was used to compare the curing behavior of the three systems under identical conditions (*r* = 1 and *β* = 20 °C/min). The results registered during the first run are presented in Figure 5.

**Figure 6:**
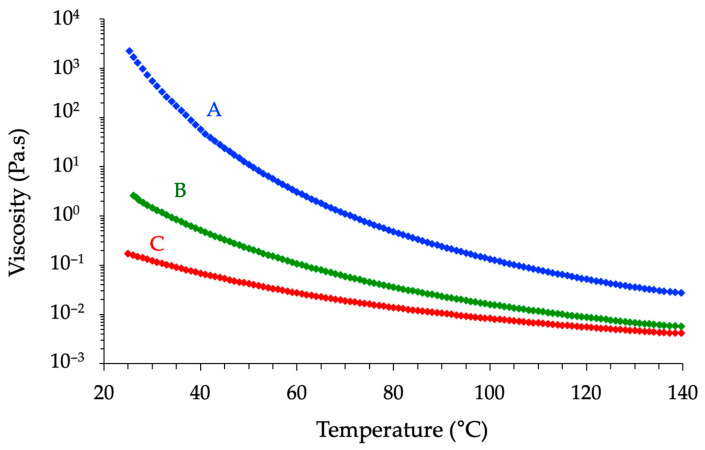
Evolution of the viscosity of each epoxy prepolymer in the temperature range between 25 and 140 °C (A = Novo, B = BADGE, C = ECy).

The exothermic peak characteristic of the curing reaction between the epoxy moieties and the anhydride units revealed a strong dependence on the molecular architecture of the epoxy precursor. Considering the temperature *Tp* at which the reaction rate is maximal, the following trend was consistently observed across the formulations: Novo < BADGE < ECy. While it could be tempting to attribute such a trend to differences in viscosity and molecular mobility, viscosity–temperature profiles measured for the neat prepolymers revealed the opposite behavior since the cycloaliphatic epoxy exhibited the lowest viscosity over the tested temperature range. In contrast, novolac prepolymer was the most viscous (Figure 6). Then, even if the differences observed decreased with increasing temperature, the molecular mobility in the uncured state cannot account for the observed differences in reactivity. Instead, the disparity in *Tp* appears to be governed primarily by the epoxy group’s chemical nature and the functionality of the prepolymer. Novolac-based epoxies are highly functionalized with multiple glycidyl ether groups per molecule, which statistically enhances the likelihood of reactive encounters, thus facilitating rapid crosslinking even at lower temperatures (Table 1). BADGE, a difunctional epoxy, forms networks with lower crosslink density and, consequently, lower reactivity. The cycloaliphatic epoxy, while difunctional as well, contains oxirane rings embedded within rigid aliphatic structures. It cures more slowly due to the lower accessibility of its epoxy groups to the curing agent units and its lower intrinsic electrophilicity compared to glycidyl ethers.

**Figure 7:**
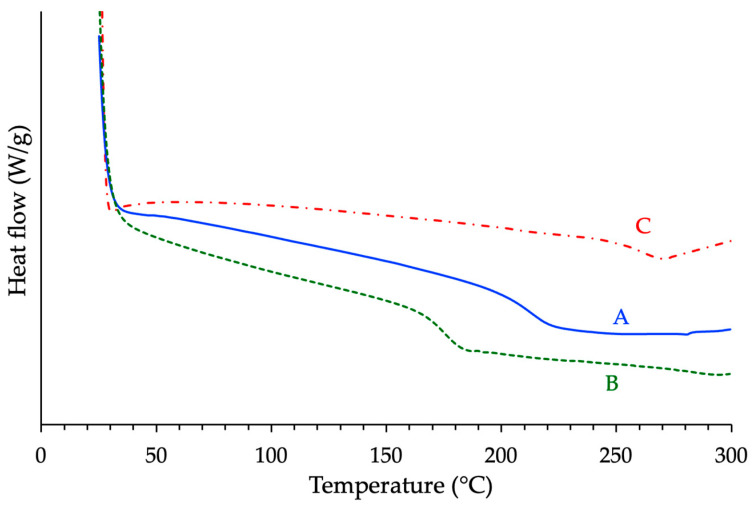
Calorimetric analysis of the different cured samples with r = 1 (A = Novo, B = BADGE, C = ECy).

Interestingly, the ranking observed for the glass transition temperatures (*Tg*) of the cured materials did not match the trend in curing reactivity. The *Tg* values increased in the order: BADGE < novolac < cycloaliphatic epoxy (Figure 7). The BADGE-based resin exhibited the lowest *Tg* (approximately 145 °C), consistent with its low crosslink density and flexible aromatic backbone. The novolac-based network, with its high degree of branching and aromatic content, showed an intermediate *Tg* value (ca. 184 °C). In contrast, the cycloaliphatic epoxy system yielded the highest *Tg* (about 253 °C), which is somewhat counterintuitive given its lower reactivity. However, this can be attributed to the rigid and symmetrical structure of the cycloaliphatic backbone, which restricts segmental motion and enhances the thermal stiffness of the network.

The *Tg* value obtained for the Novo–MNA system (184 °C) is also in very good agreement with those generally reported in the literature for comparable novolac–anhydride networks. For instance, Reynolds et al. [23] reported *Tg* values of 187–190 °C (DMA, tan δ peak) for EPN-1138 novolac/MNA thermosets designed for high-temperature composite applications, while Fedoseev et al. [24] observed similar values (178–195 °C) for epoxy–novolac systems cured with MHHPA, a slightly less rigid anhydride than MNA. Minor deviations of a few degrees can be attributed to differences in epoxy equivalent weight and molecular-weight distribution of the commercial novolac used, as also discussed by Qian et al. [25]. Variations in curing protocol and DSC heating rate can likewise cause small shifts in *Tg* [26]. Altogether, the consistency of these results confirms the reliability of the experimental procedure and the accuracy of the measured thermal properties.

A similar comparative analysis can be extended to the BADGE–MNA system. Reported glass-transition temperatures for epoxy–anhydride networks span a broad range—from about 60 °C up to 200 °C—depending on the catalyst type, anhydride/epoxy stoichiometric ratio, and epoxy functionality. For instance, Bifulco et al. measured a *Tg* of 65 °C (DSC, N_2_, 10 °C·min^−1^) for a DGEBA–MNA formulation catalyzed with 2-methylimidazole (0.5% *w*/*w*), reflecting the limited crosslink density achieved at such low catalyst loading [27]. Conversely, Bouillon et al. reported a *Tg* of 142 °C for a DGEBA–MNA network cured with 1-methylimidazole (1% *w*/*w*) at stoichiometric balance (*r* = 1), confirming that imidazole catalysis at optimized concentration promotes extensive copolymerization and higher network rigidity [28]. In comparison, Galy et al. found *Tg* values around 110–115 °C for DGEBA–MTHPA systems catalyzed by tertiary amines (BDMA, r = 1), while Montserrat et al. reported similar values for BADGE–MHHPA formulations under equivalent conditions [29,30]. The BADGE–MNA formulation investigated in the present work, catalyzed with 2.5% *w*/*w* 1-methylimidazole (*r* = 1), exhibits a *Tg* of 145 °C, consistent with the upper range of imidazole-catalyzed epoxy–anhydride thermosets. This result confirms that the selected catalyst and stoichiometric control favor efficient anhydride–epoxy copolymerization, yielding a dense and homogeneous cross-linked architecture comparable to those of high-performance BADGE-based networks reported in the literature.

Regarding the cycloaliphatic system, literature values for the glass transition temperature of cycloaliphatic epoxy–anhydride networks generally range between 220 °C and 260 °C, depending on the anhydride, accelerator, and curing schedule. Belmonte et al. reported *Tg* of about 240–250 °C for a BCDE/MHHPA network catalyzed by 1-methylimidazole [31]. Similarly, Lu et al. confirmed *Tg* values of approximately 250 °C for high-performance ECy/MHHPA thermosets, highlighting the significant contribution of the rigid alicyclic backbone to network stiffness [32]. These data are consistent with the *Tg* of 253 °C obtained for the ECy–MNA formulation studied here. Such a high glass-transition temperature mainly arises from the symmetric and conformationally constrained structure of the alicyclic rings, which significantly restricts segmental motion despite the difunctional character of the prepolymer. As emphasized by Jin et al., this behavior illustrates how molecular topology and ring rigidity can compensate for limited functionality, leading to excellent thermomechanical performance with potential suitability for high-temperature materials processing [33].

Overall, these findings highlight a crucial decoupling between the reactivity of the formulations and their final thermal properties. While curing kinetics are strongly influenced by the epoxy functionality and electrophilicity, the resulting *Tg* is more dependent on the rigidity and topology of the polymer network. This distinction is significant for high-temperature applications, where both rapid curing and elevated glass transition temperatures of the matrix are essential for efficient processing and long-term performance. Subsequent rheological analyses (Section 3.3) will further verify the consistency of the structural factors discussed above.

### 3.3. Rheological Measurements on the Different Optimized Formulations

#### 3.3.1. Kinetic Analyses

To gain deeper insights into the cure kinetics of the three epoxy/anhydride systems, isothermal rheological analyses were performed under constant temperature conditions ranging from 40 °C to 140 °C. These experiments were conducted on reactive formulations with an anhydride/epoxy ratio *r* = 1 and an optimized catalyst loading of 2.5% *w*/*w* relative to the anhydride. The evolution of the complex shear modulus (G*) over time was monitored to characterize the progression of network formation and to evaluate the times characteristic of gelation and vitrification, respectively.

All three systems exhibited the typical sigmoidal evolution of G* as a function of time, reflecting the distinct stages of the curing process—from the initial liquid state (*G*″ > *G*′), through network formation (i.e., gelation), and finally to structural stiffening associated with vitrification [6,34,35]. The attainment of a time-invariant *G*′ plateau indicates the network stabilization under the selected curing conditions corresponding to the state referred to here as “complete conversion under the chosen thermal protocol.” Nevertheless, post-curing or degradation phenomena may still occur during subsequent thermal exposures.

**Figure 8:**
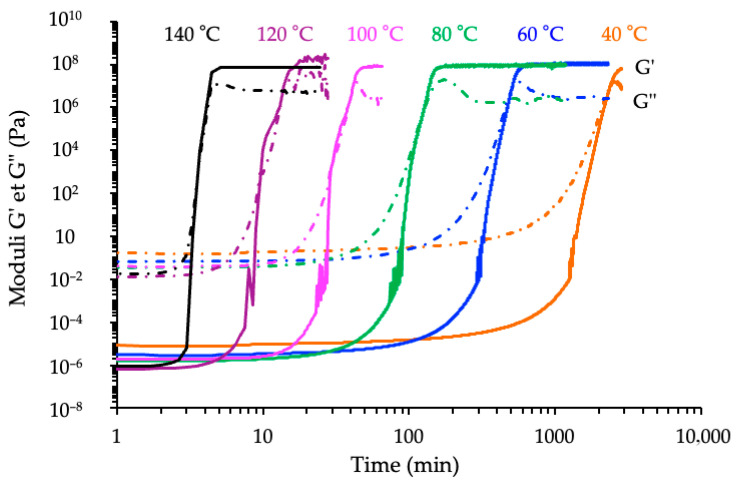
Influence of the temperature on the kinetic rheological analyses of the reactive formulation ECy-MNA-1MI 2.5% *w*/*w* with *r* = 1.

As expected, increasing the isothermal temperature led to shorter induction times and faster modulus buildup, due to enhanced molecular mobility and increased reaction rates [13]. A compilation of rheological kinetics registered with the formulation derived from the cycloaliphatic prepolymer ECy is shown in Figure 8.

Comparable temperature-dependent acceleration of curing reaction has been reported for several epoxy–anhydride systems. Ampudia et al. observed a reduction of the gel time from about 40 min at 140 °C to 10 min at 180 °C for a DGEBA–MHHPA resin [35], while Palomo et al. reported a similar trend for a cycloaliphatic epoxy–MHHPA formulation, with a gelation time decreasing from 25 min at 140 °C to less than 10 min at 160 °C [6]. Belmonte et al. further confirmed that vitrification occurred significantly earlier at higher curing temperatures in ECy–MHHPA networks [31].

**Figure 9:**
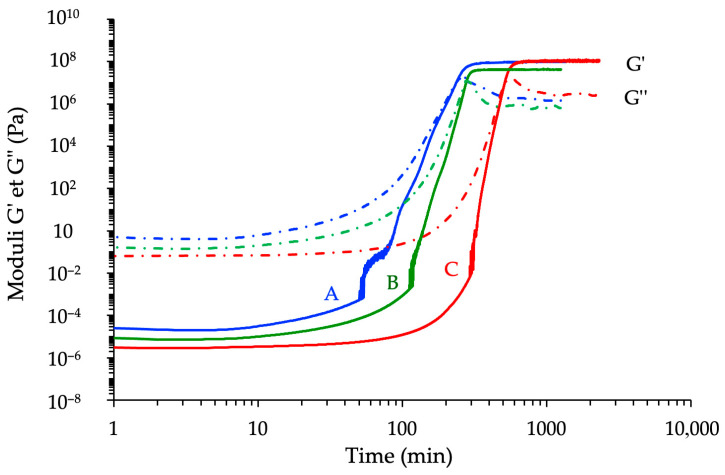
Comparison of the different rheological kinetics registered at T = 60 °C with the various reactive formulations prepared with MNA as hardener and 2.5 *w*/*w* of 1MI as catalyst (A = Novo, B = BADGE, C = ECy).

Despite this general behavior, notable differences emerged between the three reactive formulations. The novolac-based epoxy exhibited the most rapid increase in G*, with the shortest gelation time across all temperatures studied. This behavior correlates with its higher functionality and elevated reactivity, as previously observed in DSC experiments. The BADGE-based system showed intermediate kinetics, while the cycloaliphatic epoxy displayed a significantly delayed onset of modulus increase and slower network buildup, especially at lower temperatures. This hierarchy between epoxy prepolymers is shown in Figure 9 for *T* = 60 °C.

Similar kinetic behavior has been reported in the literature. Belmonte et al. [31] and Lu et al. [32] investigated cycloaliphatic epoxy formulations (ECy/MHHPA) catalyzed by imidazole. They observed slower curing compared to aromatic or novolac epoxy-anhydride systems. This trend is consistent with the lower reactivity of the ECy–MNA formulation reported here.

**Figure 10:**
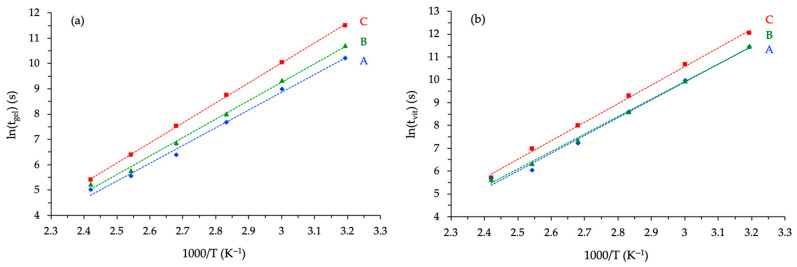
Influence of temperature on the gel time (**a**) and vitrification time (**b**) of the different epoxy formulations (A = Novo, B = BADGE, C = ECy).

These differences in reactivity between the various systems studied in the present work can also be observed by plotting the gel and vitrification times, extracted from G*(*t*), as a function of the temperature used for rheological kinetics (Figure 10). Regardless of the temperature selected, the gelation or vitrification time of the novolac prepolymer-derived formulation was significantly shorter than that characteristic of the ECy-based reaction mixture and, to a lesser extent, that produced with the BADGE prepolymer.

The Arrhenius analysis of the gel time and vitrification time curves enabled estimation of their apparent activation energies (*Ea*) for each formulation (Table 4). The BADGE and novolac systems exhibited relatively similar *Ea* values, indicating comparable temperature dependence. At the same time, the cycloaliphatic formulation showed a markedly higher *Ea*, confirming its reduced reactivity at lower temperatures and its stronger sensitivity to thermal activation. These trends demonstrate that at high temperatures (*T* > 140 °C), the differences in reactivity between the three systems studied become much smaller than at 40 °C. The differences in reactivity are even smaller at 200 °C, a representative high-curing temperature commonly employed for high-temperature epoxy–anhydride applications.

The apparent activation energies derived from DSC analyses (58–66 kJ·mol^−1^ depending on the epoxy precursor) are in excellent agreement with literature data for epoxy–anhydride systems catalyzed by imidazoles. Woo and Seferis [11] and Jain et al. [4] reported *Ea* values between 55 and 70 kJ·mol^−1^ for DGEBA and cycloaliphatic-based resins. Fedoseev et al. obtained similar values for heat-resistant epoxy–anhydride networks [24].

From a practical standpoint, these kinetic data help define the effective processing window at 200 °C for fast-curing epoxy–anhydride systems. Under such conditions, the vitrification times reported in Table 4 provide a comparative indication of how rapidly each formulation develops a solid network structure during curing. Only the novolac-based formulation met this criterion under the tested conditions. In contrast, the BADGE-based resin appears borderline, and the ECy-based system required longer times to reach the glassy state within the available thermal window.

#### 3.3.2. Correlation with Conversion Degree Determined by DSC

**Figure 11:**
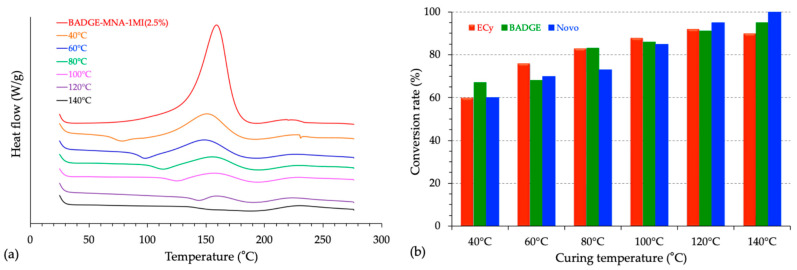
Curing progress of the epoxy–anhydride systems by DSC. (**a**) DSC thermograms of the representative BADGE–MNA formulation after partial curing at different temperatures, showing the progressive decrease of the residual exothermic peak. (**b**) Evolution of the conversion degree (α) with partial curing temperature for the three epoxy–anhydride systems.

To complement the rheological kinetic analyses, the same samples previously subjected to isothermal rheological measurements were subsequently characterized by DSC to quantify the degree of conversion (*α*) reached at each curing temperature. Indeed, such a criterion is essential in the study of thermoset resins as widely described by Sickfeld et al. for different epoxy systems [26]. The conversion was calculated as *α* = 1 − (*A*/*A*_0_), where *A*_0_ is the total reaction enthalpy of the uncured mixture and *A* is the residual exothermic area measured on the partially cured sample. Figure 11a shows the DSC thermograms of the representative BADGE–MNA formulation after the rheological kinetic tests performed at different temperatures, highlighting the progressive reduction of the residual exothermic peak as the temperature increases. The corresponding evolution of the conversion degree with curing temperature for the three epoxy–anhydride systems is presented in Figure 11b. The results demonstrate that at low temperatures (<100 °C), vitrification significantly restricts the advancement of the reaction. In contrast, above 120–140 °C, the conversion exceeds 90%, reflecting the development of an advanced network under the applied conditions. These findings are consistent with the rheological behavior previously observed, linking the progressive increase in viscoelastic moduli to the chemical conversion of the network.

#### 3.3.3. Thermomechanical Analyses of Cured Materials

To complement the isothermal rheological analyses, the thermomechanical properties of the cured materials were investigated by dynamic torsional rheometry. Using the same formulations as in the previous section—i.e., an anhydride-to-epoxy ratio *r* = 1 and a catalyst content fixed at 2.5% *w*/*w* relative to the anhydride—the three resins were thermally cured according to the protocol described in the Materials Section. The resulting materials were then subjected to dynamic torsion tests over a wide temperature range to monitor their viscoelastic behavior.

**Figure 12:**
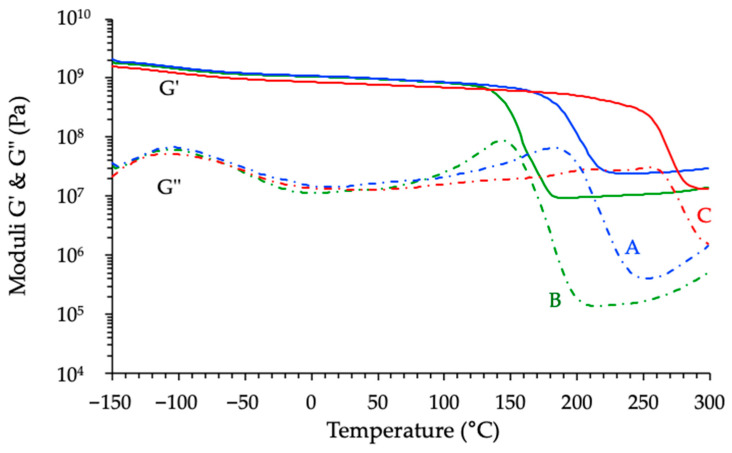
Thermomechanical profiles of the different cured materials (A= Novo-MNA, B = BADGE-MNA, C = ECy-MNA).

The corresponding results, plotted in Figure 12, showed pronounced differences between the three formulations. The temperature *Tα* associated with the peak in *G″*, which can be considered here as the glass transition temperature (*Tg*), follows the same trend as that previously observed by DSC. Indeed, the cycloaliphatic-based system exhibits the highest *Tg* (~253 °C), followed by the novolac formulation (~184 °C), and finally the BADGE-derived network (~145 °C). This hierarchy echoes the trends previously observed in DSC measurements. It supports the idea that the cycloaliphatic precursor gives rise to a network with more restricted segmental mobility at high temperature. In detail, the slight reduction of the *Tg* measured by rheometry compared to that registered by calorimetry can be explained by differences in thermal history (specific heating ramp and ultimate exposure temperature) and in sample size.

However, when considering the rubbery plateau moduli extracted from the same torsional tests, the trend did not mirror the *Tg* hierarchy. Indeed, the highest plateau modulus was observed for the novolac-based system, followed by the cycloaliphatic and BADGE-based networks. While the rubbery plateau modulus is often used as a qualitative indicator of crosslink density, it must be interpreted here cautiously. Indeed, according to rubber elasticity theory, the crosslink density *ν* (mol m^−3^) is related to the shear modulus $G_{r}^{\prime }$ in the rubbery state through the classical relation (2):

(2) $$\nu =\;\frac{G_{r}^{\prime }}{R\;T}\;$$

where *R* is the universal gas constant (8.314 J/mol/K), and *T* is the temperature (expressed in K) at which the modulus is measured (usually at *T* = *Tg* + 50) [36].

Consequently, while the relative height of the rubbery plateau can suggest differences in crosslink density between formulations, a quantitative comparison requires normalization by temperature and proper consideration of the measurement conditions. The calculated values confirmed that the novolac resin possesses the highest crosslink density, in line with its high functionality and aromatic rigidity. The cycloaliphatic and BADGE-based systems exhibit lower crosslink densities, with only a slight difference between the two (Table 5).

Then, considering these results, it is now clear that crosslink density does not correlate directly with *Tg* in these systems. Indeed, *Tg* reflects not only crosslink density but also the segmental stiffness and mobility of the polymer backbone. Therefore, the higher modulus observed for the novolac system results from a denser and more rigid network structure, consistent with the multifunctionality and aromatic rigidity of the novolac epoxy precursor. Nevertheless, this interpretation remains semi-quantitative as the rubber elasticity theory used before assumes that the material behaves in an ideally elastic manner above *Tg*, which is not always the case for highly crosslinked resins [37,38].

**Figure 13:**
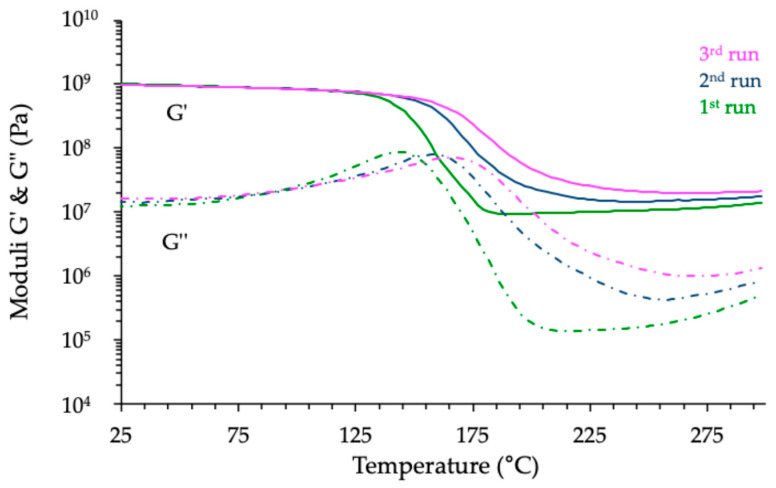
Successive thermomechanical analyses of the cured BADGE-MNA-1MI material (focus on the temperature range between 25 and 300 °C).

**Figure 14:**
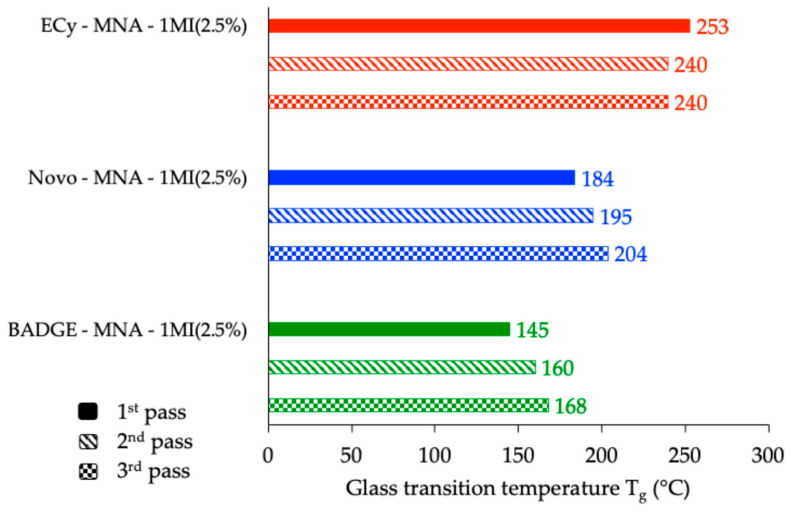
Evolution of *Tg* of different crosslinked materials after consecutive thermomechanical analyses.

A closer examination of the thermomechanical profiles shown in Figure 12 reveals a slight increase in the storage and loss moduli beyond the glass transition region, especially for the novolac- and BADGE-based networks. This phenomenon, occurring well into the rubbery plateau, suggests that the polymer network might still be evolving at elevated temperatures. Such behavior prompted further investigation, as it could result either from an incomplete cure—leading to post-curing phenomena—or from the onset of thermal degradation mechanisms. To assess the nature of this evolution, two additional temperature sweeps were performed on each cured sample under identical experimental conditions, aiming to evaluate the thermal stability and structural maturity of the networks [39]. Figure 13 shows, as an example, the results registered with the material derived from BADGE, while Figure 14 provides a compilation of the evolution of the *Tg* of the different materials induced by the various thermomechanical analyses.

The repeated thermomechanical analyses provided further insights into the structural evolution of the cured resins under thermal solicitation. For the BADGE-based system, the *Tg* increased from 145 °C during the first run to 168 °C after the third, indicating that post-curing reactions were still occurring and contributing to further crosslinking under the applied heating schedule. A similar trend was observed for the novolac-based network, where the *Tg* increased from 184 °C to 204 °C, but with a smaller relative difference. These results suggest that, despite the curing protocol applied, the network structures of these two systems were not fully matured and continued to evolve upon heating.

**Figure 15:**
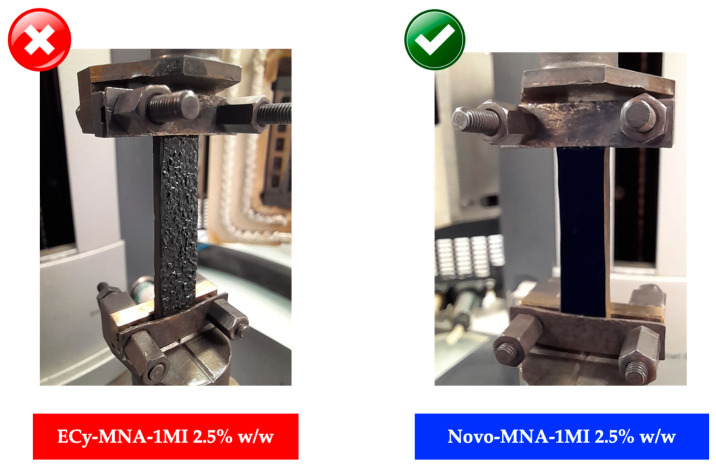
Overview of cured epoxy samples based on ECy (**left**) and Novo (**right**) after three successive thermomechanical analyses.

In contrast, the ECy-based material exhibited a different behavior: its *Tg* decreased slightly, from 253 °C to 240 °C over the three runs, pointing to possible thermal degradation rather than additional crosslinking. This assumption seemed further supported by the physical appearance of the samples after testing. While the BADGE and novolac specimens maintained structural integrity, the ECy-based sample showed the formation of blisters and, in some cases, the emergence of surface cracks, highlighting its limited thermal stability under high-temperature exposure (Figure 15). These photographs are provided solely as qualitative illustrations of the macroscopic appearance of the samples after thermomechanical testing and are not intended as microscopic or quantitative analyses. The origin of this degradation will be discussed in Section 3.4.

To complete the thermomechanical characterization of the cured systems and to gain further insight into their high-temperature performance, thermogravimetric analyses (TGA) were undertaken. These tests aimed to provide quantitative data on the thermal stability of the three networks, thereby complementing the previous observations made during temperature sweep experiments.

### 3.4. Thermogravimetric Experiments

#### 3.4.1. Determination of the Apparent Degradation Temperature (5 °C/min)

**Figure 16:**
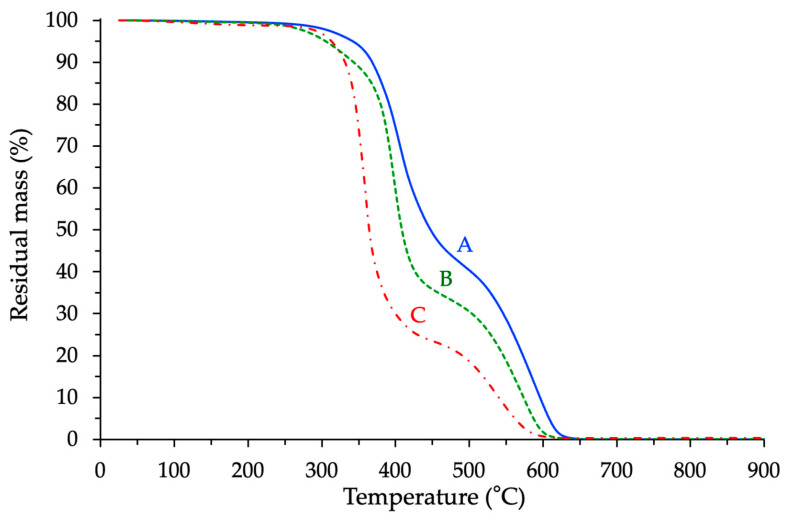
Thermogravimetric profiles of the different cured materials (A = Novo-MNA, B = BADGE-MNA, C = ECy-MNA).

Thermogravimetric analyses were first carried out under dynamic conditions, using a constant heating rate of 5 °C/min. The onset of thermal degradation was estimated by determining the temperature corresponding to a 10% weight loss (=*T*_10_). This threshold, while widely used in the literature as a pragmatic indicator of thermal stability, remains somewhat arbitrary, and the degradation temperature should be named as “apparent”. Indeed, it is sensitive to both the selected weight-loss percentage and the heating rate [40,41]. But, for the same heating ramp, it is possible to evaluate and compare different systems. Within the above parameters, significant differences emerged among the three cured systems (Figure 16). The novolac-based resin exhibited the highest apparent thermal stability, with the highest degradation temperature (*T*_10_ = 368 °C), while the formulation derived from the cycloaliphatic epoxy showed the lowest one (*T*_10_ = 331 °C). The aromatic system based on BADGE occupied an intermediate position (*T*_10_ = 343 °C). These results underline the strong influence of the epoxy prepolymer structure on the initial thermal degradation behavior of the cured networks.

#### 3.4.2. Isothermal TGA Analyses at T = 250 °C

**Figure 17:**
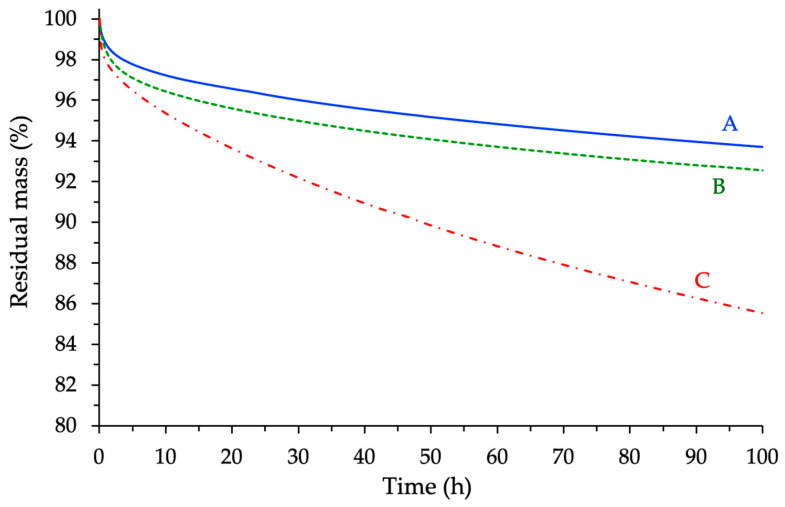
Isothermal TGA analyses of the different cured materials recorded at T = 250 °C (A = Novo-MNA, B = BADGE-MNA, C = ECy-MNA).

To complement the dynamic TGA results and better evaluate the long-term thermal endurance of the cured systems, additional thermogravimetric analyses were conducted under isothermal conditions. A temperature of 250 °C was selected as a discriminating condition, allowing for meaningful comparison of the degradation behavior over a relatively short duration (100 h). The results confirmed the trends previously observed. Indeed, the cured network derived from the cycloaliphatic epoxy exhibited the highest mass loss, with only 85.5% of the initial mass retained after the test (Figure 17). In contrast, the novolac-based system displayed the highest mass retention (93.7%), while the BADGE-based resin showed intermediate performance (92.6%). These findings are entirely consistent with the thermal ranking established under dynamic conditions, as well as with the trends identified from the thermomechanical analyses and the visual inspection of the materials post-rheological tests.

**Figure 18:**
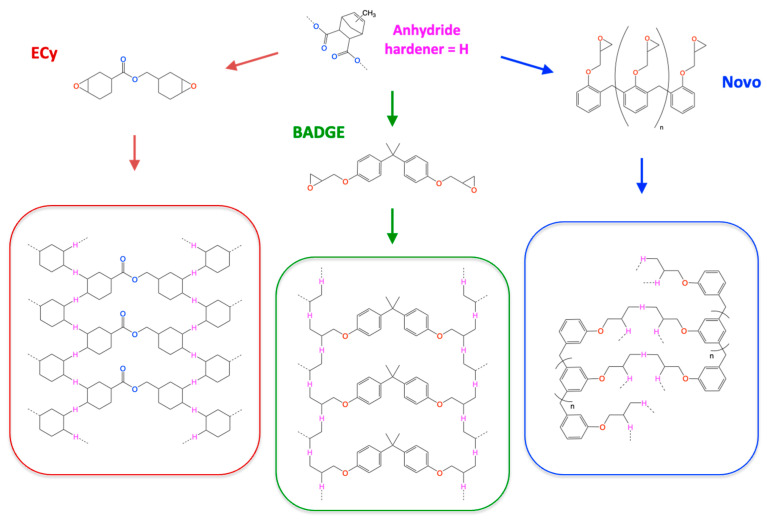
Schematic representation of the different epoxy networks produced from the respective reaction of each epoxy prepolymer with MNA.

This hierarchy in thermal resistance can be interpreted through a deeper structure–property relationship analysis, considering both the architecture of the epoxy precursors and the resulting network characteristics, as represented in Figure 18.

First, the superior thermal stability of the novolac-based system can be primarily attributed to its highly aromatic structure and elevated epoxy functionality, as already described in the literature [42,43]. The phenolic novolac backbone, densely substituted with glycidyl ether groups, leads to a highly crosslinked network upon curing. This high crosslinking density, already evidenced by the elevated rubbery plateau modulus and confirmed quantitatively via rheological estimation of the crosslink density, contributes to the formation of a thermally robust structure, more resistant to bond cleavage and chain scission at elevated temperatures. The presence of numerous aromatic rings also adds intrinsic thermal resilience due to the high bond dissociation energies of aromatic systems.

The present findings are in very good agreement with earlier reports on novolac–anhydride systems. Classical studies by Freeman and Becker [42] and Gac et al. [43] on MNA-cured novolac epoxies showed that degradation typically begins between 250 °C and 280 °C, initiated by ester bond cleavage followed by oxidation of the aromatic backbone. At the same time, Fleming identified the same homolytic C–O bond scission as the main degradation pathway [44]. More recent work by Fedoseev et al. confirmed limited mass losses (<10%) below 300 °C for epoxy–novolac systems cured with MNA or related anhydrides [24]. In the present study, the Novo–MNA resin retained approximately 94% of its initial mass after 100 h at 250 °C, thereby positioning its stability at the upper end of the literature data and confirming the high thermo-oxidative resistance of its aromatic, densely crosslinked network.

In contrast, the cycloaliphatic-based formulation, despite displaying the highest initial glass transition temperature, shows the lowest thermal stability under both dynamic and isothermal conditions. This behavior is in line with earlier studies on cycloaliphatic epoxy–anhydride systems. Belmonte et al. and Lu et al. reported *T*_10_ values around 340–345 °C for imidazole-catalyzed ECy/MHHPA networks measured under nitrogen and at heating rates of 10–20 °C·min^−1^ [31,32]. In the present work, the ECy–MNA resin exhibits a lower *T*_10_ of 331 °C, which can be attributed not only to its specific molecular structure but also to the more severe testing conditions used here (air atmosphere, 10 °C·min^−1^). Under such conditions, oxygen diffusion and prolonged thermal exposure accelerate degradation, leading to lower apparent onset temperatures. The influence of atmosphere and heating rate on the measured degradation temperature has been widely documented for polymers of very different natures [40,41,45]. Several structural factors further account for this behavior. The aliphatic, non-aromatic nature of the epoxy precursor reduces the inherent thermal stability of the resulting network, since C–C bonds within alicyclic units are more prone to thermally induced cleavage than their aromatic counterparts [32]. Moreover, the presence of ester linkages adjacent to the cycloaliphatic rings introduces thermally labile sites (Figure 18) that are particularly sensitive under oxidative environments [31,46]. Although the ECy-based system shows a high *Tg* and a reasonable crosslink density, this does not necessarily ensure a thermally resistant network: *Tg* reflects chain rigidity, whereas thermo-oxidative stability depends on bond chemistry and degradation pathways [44]. The significant mass loss observed in isothermal TGA and the visible damage (blistering, cracking) after successive rheological scans further confirm the susceptibility of this network to ester bond scission and backbone fragmentation, in agreement with the degradation mechanisms proposed for alicyclic epoxy–anhydride thermosets.

As for the BADGE-based material, it presents an intermediate case. Its bisphenolic structure provides a reasonable level of aromatic content and rigidity, contributing to moderate thermal stability [47,48]. However, its lower functionality compared to novolac (only two epoxy groups per molecule) results in a network of lower crosslinking density, making it more prone to thermal softening or degradation than the novolac-derived network. This interpretation is supported by the degradation data reported for BADGE-based anhydride systems. Bifulco et al. measured *T*_10_ at about 317 °C for a BADGE–MNA formulation catalyzed with 0.5% *w*/*w* 2-methylimidazole under nitrogen and at 10 °C·min^−1^ [27], while Bouillon et al. (1989) obtained comparable values for imidazole-cured systems and slightly lower ones for tertiary-amine formulations [28]. Similarly, Yang et al. [49] and Li et al. [50] reported degradation onsets ranging from 260 to 300 °C for BADGE–anhydride thermosets catalyzed by DMP-30 or BDMA, independently of the anhydride used (MNA, MHHPA, or MeTHPA). In the present work, the BADGE–MNA material exhibits a *T*_10_ value consistent with these data, confirming its intermediate position between the thermally robust novolac-based network and the less stable cycloaliphatic analog.

These observations confirm that thermal endurance in epoxy–anhydride systems is mainly governed by the crosslinking density, the aromatic character of the network, and the chemical stability of the ester linkages formed during curing, emphasizing the importance of resin selection when designing high-temperature-resistant epoxy matrices.

These results also highlight that a high glass transition temperature does not necessarily imply superior thermal endurance. The ECy–MNA system illustrates this distinction particularly well: despite its high *Tg*, the presence of ester linkages and its moderate crosslink density facilitates oxygen diffusion, thereby accelerating thermo-oxidative degradation. Cycloaliphatic epoxy–anhydride resins are indeed often reported as thermally stable in the literature under inert atmosphere, but generally under milder conditions (below ≈ 180 °C). Under the much more severe exposures explored here (250–300 °C), oxidation and bond cleavage become the predominant processes.

Overall, the combined rheological and thermogravimetric analyses provide a complementary insight into the behavior of these resins under elevated-temperature conditions. At a representative processing temperature of 200 °C, the novolac-based formulation exhibits the fastest network formation and the highest thermal endurance, as evidenced by its limited mass loss after 100 h at 250 °C—a condition deliberately selected to discriminate long-term stability among the systems. The BADGE-based system shows intermediate curing kinetics and moderate stability, whereas the cycloaliphatic resin, despite its high initial *Tg*, exhibits slower crosslinking and reduced thermal retention. These correlations between structure, cure dynamics, and thermal stability allow meaningful comparison of the different epoxy–anhydride networks and help identify structure–property trends relevant to high-temperature applications.

**Figure 19:**
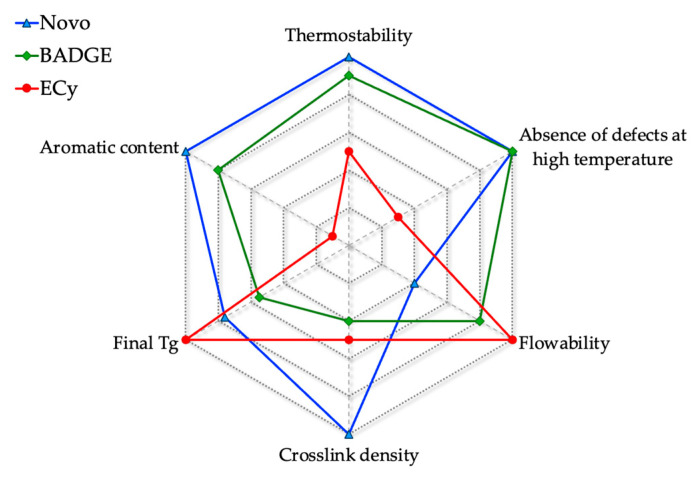
Radar plot comparing the three epoxy/anhydride systems in terms of processing and thermal performance criteria.

Selecting the most suitable epoxy prepolymer is not straightforward, as numerous interdependent factors must be considered simultaneously. To provide a more synthetic overview, the three optimized epoxy/anhydride systems were compared using a radar diagram (Figure 19). This representation emphasizes the contrasted profiles of the different structures: the novolac resin exhibits the highest crosslink density and thermostability, but suffers from limited flowability, which complicates its processing at lower temperatures. Moreover, its final *Tg* does not authorize applications where a high mechanical rigidity is required above 200 °C. Conversely, cycloaliphatic prepolymer offers excellent fluidity. After reacting with the anhydride hardener, it also provides a crosslinked material with a high *Tg*, but the latter has insufficient long-term thermal resistance. Bis-aromatic BADGE occupies an intermediate position, combining moderate processability and balanced final properties despite a lower *Tg*. In fact, this radar chart shows that no formulation appears universally optimal, although a novolac prepolymer-based formulation seems most promising if the initial viscosity issue can be resolved.

## 4. Conclusions

This study investigated the influence of epoxy prepolymer structure—novolac, cycloaliphatic, and bisphenol-A diglycidyl ether (BADGE)—on the curing behavior, thermomechanical properties, and thermal stability of epoxy/anhydride systems relevant to high-temperature resin design. As a preliminary step, each formulation was optimized with respect to anhydride-to-epoxy ratio and catalyst content to ensure balanced reactivity and crosslinking potential, thus providing a consistent basis for comparison.

The results highlighted clear contrasts among the three systems. The novolac-based formulation exhibited the highest reactivity, the highest rubbery plateau modulus, and the highest apparent thermal stability, reflecting its high functionality and aromatic backbone. The BADGE-based resin displayed intermediate performance across all metrics. The cycloaliphatic system cured more slowly, providing a broader curing window but at the expense of crosslink density and long-term thermal resistance, consistent with its aliphatic structure and ester functionalities.

The work thus provides a quantitative benchmark unifying DSC, rheological, and thermogravimetric analyses under identical catalytic and stoichiometric conditions—a contribution expected to serve as a reference for future modeling and formulation studies of epoxy–anhydride systems.

From a processing standpoint, these findings underline the trade-offs inherent to the formulation of epoxy–anhydride systems for high-temperature use. Novolac resins deliver superior thermal performance, but their high viscosity restricts low-temperature processing and narrows the workable window, with a risk of premature gelation during heating. Conversely, cycloaliphatic resins offer low viscosity and extended handling times but require higher cure temperatures or longer heating durations to achieve comparable conversion. BADGE resins represent an intermediate solution, combining moderate viscosity with balanced performance and thermal resistance.

Overall, tailoring the resin chemistry for high-temperature applications requires balancing reactivity, processability, and thermal performance, while accounting for viscosity constraints during curing. One potential avenue for future work is the development of hybrid formulations that combine the favorable attributes of different epoxy prepolymers—for example, leveraging the stiffness and thermal stability of novolac with the improved flow and processing latitude of cycloaliphatic or BADGE systems. Such approaches could yield more versatile resin systems for thermally resistant epoxy matrices while remaining consistent with the fundamental structure–property relationships established in this work. Although blends of BADGE and novolac epoxies have been reported in the literature, a comparison with our systems remains non-trivial due to differences in epoxy equivalent weights and the use of non-anhydride hardeners [51,52,53,54].

## Figures and Tables

**Table 1:** Details about the different chemicals employed in this research.

Common Name in This Study	Abbreviation	CAS	Chemical Structure
Cycloaliphatic epoxy resin	ECy	2386-87-0	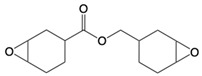
Novolac epoxy resin	Novo	28064-14-4	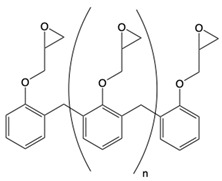
Linear diaromatic epoxy	BADGE	1675-54-3	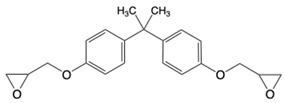
Cyclic anhydride	MNA	25134-21-8	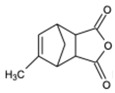
Catalyst	1MI	616-47-7	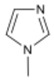

**Table 2:** Data issued from the exploitation of the first and second DSC thermograms registered with the different formulations with variable *r* values.

Formulation	*r*	*Tp* (°C)	Δ*H* (J/g)	*Tg* (°C)
ECy-MNA-1MI 5% *w*/*w*	0.7 0.8 1 1.2	171 172 173 172	327 338 350 335	206 212 250 234
Novo-MNA-1MI 5% *w*/*w*	0.7 0.8 1 1.2	156 156 156 157	384 366 354 312	175 183 178 175
BADGE-MNA-1MI 5% *w*/*w*	0.7 0.8 1 1.2	162 162 159 159	398 363 320 303	139 151 148 152

**Table 4:** Exploitation of the Arrhenius dependence of gel and vitrification times.

Prepolymer Used in the Formulation	Gelation Time		Vitrification Time	
	Activation Energy (kJ/mol)	Value—T = 200 °C (s)	Activation Energy (kJ/mol)	Value—T = 200 °C (s)
Novo	58.2	14	64.9	20
BADGE	60.7	16	64.0	22
ECy	65.7	20	67.8	29

**Table 5:** Exploitation of the thermomechanical results for the three cured materials.

Cured Formulation (*r* = 1)	*Tg* (°C)	$G_{r}^{\prime }$ (MPa)	*ν* (mol m^−3^)
ECy-MNA-1MI 2.5% *w*/*w*	253	13.2	2768
Novo-MNA-1MI 2.5% *w*/*w*	184	24.5	5808
BADGE-MNA-1MI 2.5% *w*/*w*	145	9.5	2418

## Data Availability

The data presented in this study are available on request from the corresponding author as they belong primarily to a collaborative project.

## Abbreviations

The following abbreviations are used in this manuscript:

AEW	Anhydride Equivalent Weight
BADGE	Bisphenol A Diglycidyl Ether
BDMA	Benzyl dimethylamine
CAS	Chemical Abstracts Service
DMP-30	Tris (dimethylaminomethyl) phenol
DSC	Differential Scanning Calorimetry
ECy	Cycloaliphatic epoxy prepolymer
EEW	Epoxy Equivalent Weight
MHHPA	Methylhexahydrophthalic Anhydride
MTHPA	Methyltetrahydrophthalic Anhydride
MNA	Methyl Nadic Anhydride
Novo	Novolac epoxy prepolymer
TCI	Tokyo Chemical Industry
TGA	Thermogravimetric analysis
*Tp*	Peak temperature
*Tg*	Glass transition temperature
T_10_	Degradation temperature related to a mass loss of 10% *w*/*w*
1MI	1-methylimidazole
